# Cell Walls and the Developmental Anatomy of the *Brachypodium distachyon* Stem Internode

**DOI:** 10.1371/journal.pone.0080640

**Published:** 2013-11-21

**Authors:** Dominick A. Matos, Ian P. Whitney, Michael J. Harrington, Samuel P. Hazen

**Affiliations:** 1 Biology Department, University of Massachusetts, Amherst, Massachusetts, United States of America; 2 Molecular and Cellular Biology Graduate Program, University of Massachusetts, Amherst, Massachusetts, United States of America; Lawrence Berkeley National Laboratory, United States of America

## Abstract

While many aspects of plant cell wall polymer structure are known, their spatial and temporal distribution within the stem are not well understood. Here, we studied vascular system and fiber development, which has implication for both biofuel feedstock conversion efficiency and crop yield. The subject of this study, *Brachypodium distachyon*, has emerged as a grass model for food and energy crop research. Here, we conducted our investigation using *B. distachyon* by applying various histological approaches and Fourier transform infrared spectroscopy to the stem internode from three key developmental stages. While vascular bundle size and number did not change over time, the size of the interfascicular region increased dramatically, as did cell wall thickness. We also describe internal stem internode anatomy and demonstrate that lignin deposition continues after crystalline cellulose and xylan accumulation ceases. The vascular bundle anatomy of *B. distachyon* appears to be highly similar to domesticated grasses. While the arrangement of bundles within the stem is highly variable across grasses, *B. distachyon* appears to be a suitable model for the rind of large C_4_ grass crops. A better understanding of growth and various anatomical and cell wall features of *B. distachyon* will further our understanding of plant biomass accumulation processes.

## Introduction

Grasses emerged 70 to 55 million years ago with numerous distinctions from eudicotyledons and other monocotyledons including unique vascular patterning and internal anatomy [1], [2]. In the grass stem, vascular tissue is organized in an atactostele pattern with vascular bundles scattered throughout the ground tissue and the phloem outside of the xylem [1]. The sieve elements and their companion cells are generally smaller than their xylem counterparts. Within the xylem, the vessel elements typically are larger and have thicker cell walls than tracheids [3]. The area between the vascular bundles, the interfascicular region, may be comprised of two different cell types: parenchyma and sclerenchyma [1]. Parenchyma cells typically have a large central vacuole to facilitate storage of water, starch and other small molecules. These very large cells are predominantly found in the pith although in some species they can be found in vascular bundles and in the interfascicular region [3]. Parenchyma cells remain alive throughout the life cycle of the living plant. Sclerenchyma cells undergo cell death following secondary cell wall biosynthesis and provide mechanical support. In grasses, sclerenchyma fibers form the bundle sheath, a layer of protective fibers that surround the vascular bundle and separate phloem from xylem [1]. Collenchyma cells have thick primary cell walls and provide support to the stem [4]. In grasses, these cells tend to be absent although the cortex may still refer to layers of ground tissue found just below the epidermis and above the outermost vascular bundle. Chlorenchyma cells can also be found in grasses and are characterized as having chloroplasts and thin primary cell walls. These photosynthetic cells tend to be located near the epidermis when present.

A defining aspect of plant cell function is the wall. Semi-permeable primary walls are formed during cell elongation. Once a cell has taken final shape, some specialized cell types, which include tracheary elements and sclerenchyma cells, undergo further wall thickening inside the primary wall by secondary cell wall biosynthesis [5]. Cell walls are mostly comprised of five different components: cellulose, hemicellulose, pectin, lignin, and proteins. Cellulose is often the predominant cell wall polysaccharide. It exists as an unbranched chain containing up to 15,000 β-1,4-linked glucose residues [6]. The glucan chains are cross-linked to each other by hydrogen bonds and in turn are thought to spontaneously assemble to form cellulose microfibrils that provide tremendous tensile strength to plant cell walls and can exist in crystalline, para-crystalline, and non-crystalline states [7]. In contrast, the shorter hemicelluloses and pectins are chemically and physically more complex and their compositions vary among species, tissues and cell types within an individual plant [8], [9]. Commelinid monocotyledons, including the cereals, have little pectin, large amounts of glucuronoarabinoxylan and the hemicellulose mixed linkage (1,3;1,4)-β-glucan [8], [10]. Lignin is a phenolic polymer built from three monolignols: *p*-coumaryl, coniferyl, and sinapyl alcohols that polymerize to form *p-*hydroxyphenyl, guaiacyl, and syringyl phenylpropanoid units [11]. Crosslinking lignin with hemicellulose in secondary cell walls of vascular tissue increases hydrophobicity and thus gives these functional tissues the capacity to efficiently conduct water [12]. Lignin contributes to the structural rigidity needed to keep the plant continuously erect as it grows.

Plant cell wall polysaccharides can be saccharified and fermented by some microorganisms that make byproducts capable of functioning as fuel [13]. Therefore, the biosynthesis of plant cell walls and the relative efficiencies with which they can be converted to sources of energy is of keen interest. Directly working with cultivated species or emerging bioenergy crops would be ideal, but several attributes make them challenging subjects. In general, food and energy crops are large, requiring considerable space for cultivation, and have relatively long life cycles. Crops also tend to have large and redundant genomes [14]. Recently, *Brachypodium distachyon* has emerged as a model species for various food and bioenergy crops [15], [16]. It exhibits most of the model system properties of *Arabidopsis thaliana*, but as a grass, *B. distachyon* serves as a model for potential energy crops such as *Panicum vergatum*, *Sorghum bicolor*, and *Miscanthus sp.*, as well as for the cereal crops that constitute a large part of human caloric intake [17]. Here, we investigate the accumulation of lignin, cellulose, and xylan during vascular development and describe the internal anatomy of the *B. distachyon* stem internode.

## Materials and Methods

### Plant material

Dry seeds of *B. distachyon* accession Bd21-3 were imbibed and stratified in a wet paper towel at 6 °C for seven days. Seeds were then sown in 10 cm pots containing potting mix (#2; Conrad Fafard Inc., Agawam, MA, USA). Growth chamber temperature was maintained at 20 °C with 20 h light:4 h dark cycles at a fluence rate of 220 µmol of photons^.^m^−2.^s^−1^ and relative humidity of 67 to 69. For further histochemical analysis, the first internode of the tallest stem was removed from plants of three different stages. The first stage, elongation, corresponded to when the first internode above the crown of the tallest stem was elongating. The second stage, inflorescence emergence, was when the first internode was completely elongated and the inflorescence began to emerge from the flag leaf. The third stage, senescence, was when the first internode had senesced and the stem reached its maximum height with all of the leaves showing signs of senescence.

### Whole plant measurements

Tiller count (n  =  20) was recorded as the number of stems per plant and height (n  =  25) as the tallest tiller per plant to the tip of the inflorescence of fully senesced plants. Biomass accumulation was quantified as the fresh weight (n  =  25) of the total above ground biomass. Internode length (n  =  13-21) was determined by removing the leaf sheath and imaging the tissue using a stereo dissecting Leica MZ16 F microscope (Leica Microsystems, Buffalo Grove, IL, USA) and the line measurement feature of ImageJ [18].

### Histochemistry

Approximately 200 µm transverse sections of the first internode were made manually using a No. 11 scalpel blade and a Nikon SMZ445 dissecting microscope (Nikon, Melville, NY, USA). Sections were transferred to 1.5 mL Eppendorf microcentrifuge tubes containing distilled water. Cross-sections were subjected to two different histological stains: toluidine blue or the Wiesner reagent. Following a 30 s treatment with toluidine blue (7.4 µM in H_2_O), sections were rinsed twice with distilled water. To stain and visualize lignin, sections were treated with the Wiesner reagent (79 µM phloroglucinol-ethanol in 13.7 mM HCl) for 2 min. Stained cross-sections were mounted on microscope slides and visualized using a Nikon Eclipse E200MV R microscope (Nikon, Melville, NY, USA) attached to a PixeLINK 3 MP camera (PixeLINK, Ottawa, Canada). Images were captured using the associated PixeLINK uSCOPE software (PixeLINK, Ottawa, Canada) and further processed with Adobe Photoshop CS5.5 (Adobe, Waltham, MA, USA). The cellulose-binding module CBM3a (PlantProbes, Leeds, England) was used to detect crystalline cellulose [19]. Stem cross-sections were rinsed twice with phosphate-buffered saline (PBS; 33 mM Na_2_HPO_4_, 1.8 mM NaH_2_PO_4_ and 140 mM NaCl, pH 7.2) and suspended for 30 min in blocking buffer (PBG; 0.2% fish gelatin and 2.5% Bovine Serum Albumin in PBS, pH 7.4) prior to incubation with 100 µL of 10 µg/mL CBM3a diluted in PBG for 1 h. The solution was then removed, and two 5 min long washes were followed by two rapid changes of PBS. The cross-sections were then treated with 100 µL of anti-His antibody produced in mouse (Sigma-Aldrich, St. Louis, MO, USA) and diluted in PBG at a 1∶1000 ratio for 1 h. The solution was then removed and two 5 min long washes followed by two rapid changes of PBS. The cross-sections were then incubated with 100 µL of rabbit anti-mouse antibody conjugated to Texas Red fluorophore (Invitrogen, Grand Island, NY, USA) and diluted in PBG at a 1∶100 ratio for 1 h. Afterwards, the solution was removed and two 5 min washes were followed by two changes of PBS. A similar protocol was used to label the sections with LM10, a rat monoclonal antibody that detects xylan [20]. LM10 was diluted in PBG at a 1∶10 ratio and the secondary antibody, a goat anti-rat antibody conjugated to Texas Red fluorochrome, was diluted in PBG at a 1∶100 ratio. No tertiary antibody was required for the detection of the LM10 xylan epitope. All sections were mounted using Fluoromount Aqueous Mounting Medium (Sigma-Aldrich, St. Louis, MO, USA). Fluorescence microscopy was performed using a Leica MZ16 F microscope equipped with a mercury bulb attached to a Leica DFC300FX 1.4 MP camera (Leica Microsystems, Buffalo Grove, IL, USA). The violet filter (425 nm) was used to visualize lignin autofluorescence, and the Texas Red filter (560 nm) was used to visualize crystalline cellulose and xylan. Images were captured using the Image-Pro Plus imaging software (Media Cybernetics, Bethesda, MD, USA) and further processed using Adobe Photoshop CS5.5. Image acquisition was performed so that nominal autofluorescence was observed when analyzing unlabeled sections under Texas Red filter (Fig. S1).

### Image analysis and morphological measurements

ImageJ was used to analyze images by automatically measuring selected areas of interest after scale calibration [18]. Stained whole stem images were used to observe total vascular bundle count. To measure stem cross-section (n  =  20) and vascular bundle area (n  =  7–10 plants, 1–5 bundles/plant), the ImageJ freehand selection tool was used to trace the appropriate anatomy. For interfascicular region measurements (n  =  7–10 plants, 1–5 bundles/plant), the area in between vascular bundles was traced using the polygon tool. The walls of vessels and four adjacent bundle fibers were measured to estimate vascular bundle wall thickness (n  =  6 plants, 5 bundles/plant, 4 measurements/bundle). For interfascicular region cell wall thickness (n  =  6 plants, 5 bundles/plant, 4 measurements/bundle), lines were drawn across the adjacent cell walls of the first and second row of cells outside the bundle sheath of a vascular bundle. The adjacent walls of the second and third row of cells were also measured. Fluorescence was measured as the average pixel intensity relative to the background (n  =  5 plants, 5 sections/plant, 1–2 measurements/section). Selection tools were used to trace vascular bundles, interfascicular regions, whole stem cross-sections, and background regions. The initial images were further processed and false-colored using Photoshop CS5.5.

### Fourier transform infrared (FTIR) spectroscopy

Stem tissue was pulverized with 6.35 mm stainless steel beads (BioSpec, Bartlesville, OK) using a Retsch Mixer Mill MM400 for 15 min and dried overnight at 37°C. Three biological replicates of each growth stage were individually placed on a sodium chloride compressed slide (New Era, Cat. No. 603A03). Ten randomly selected areas were scanned for each sample by FTIR-spectroscopy. Infrared spectra were obtained on a Hyperion 3000 microscope with a Vertex 70 Bruker spectrometer and processed using OPUS 7.2 spectroscopy software. Spectra were obtained by averaging 48 scans from 829 to 1829 cm^−1^ at a resolution of 1 cm^−1^ and corrected for background absorbance by subtraction of the spectrum of the empty NaCl compressed stage. For each developmental stage, the average line spectra were calculated following post-processing normalization.

### Statistical Analysis

For each measurement, 6 to 25 independent plants were sampled. Analysis of variance and Tukey’s contrasts were performed in R v2.15.0.

## Results

### Anatomy and development of Brachypodium distachyon stem internodes

**Figure 1:**
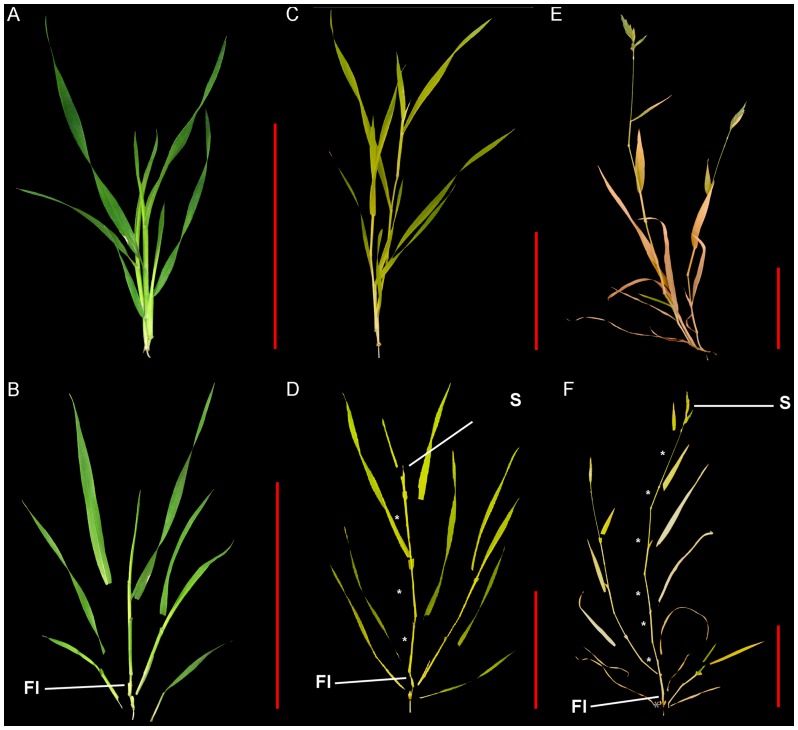
Three developmental stages selected for internode characterization in *Brachypodium distachyon*. (**A, C, E**) Intact and (**B, D, F**) dissected plants when (**A-B**) the first internode was elongating, (**C-D**) the first inflorescence was emerging, and (**E-F**) the first internode was senesced. FI, first internode; S, spike inflorescence. Scale bars (in red)  =  5 cm. Distal internodes are marked with an asterisk.

**Figure 2:**
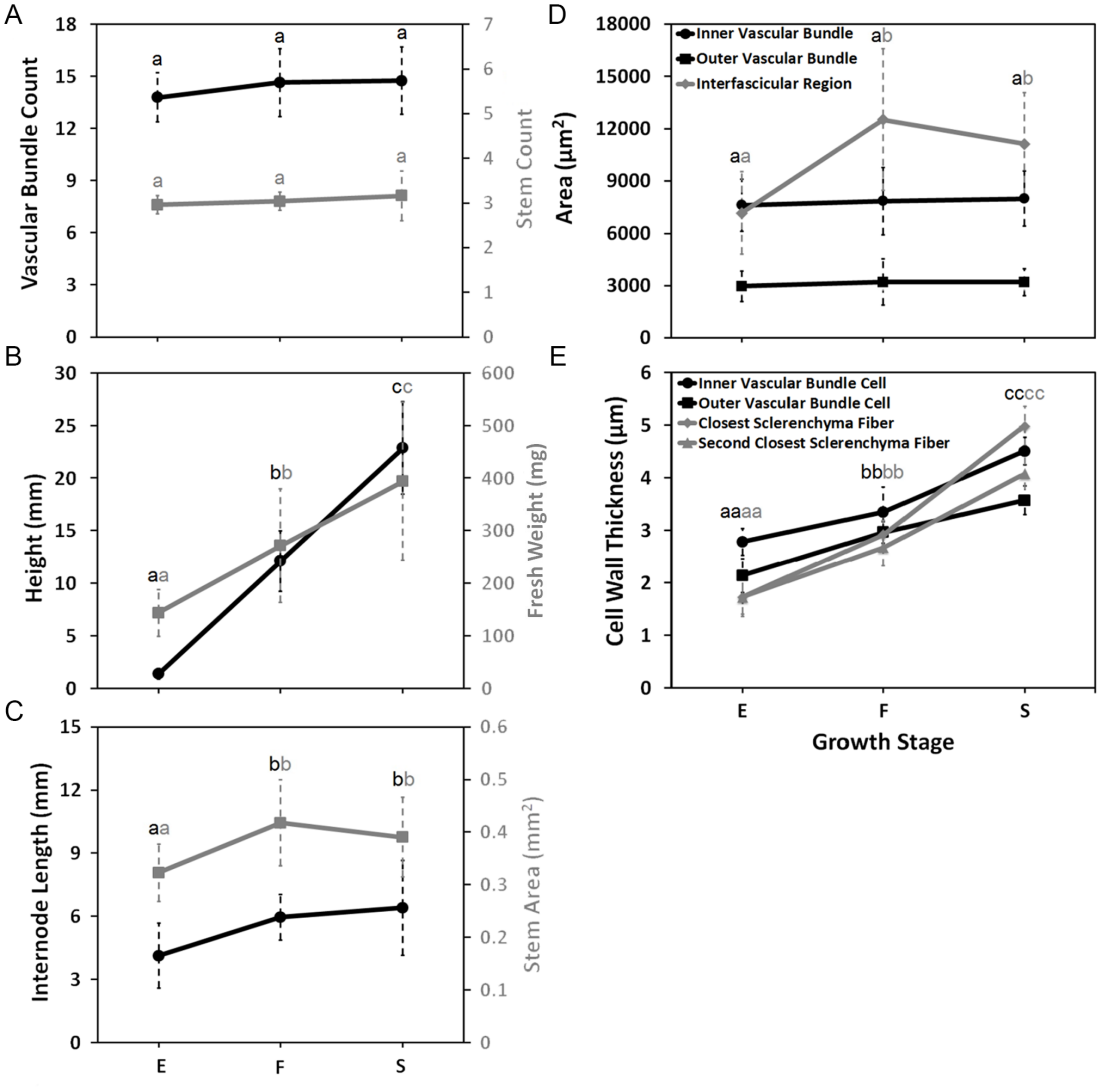
Analysis of *Brachypodium distachyon* stem internode development. (**A**) Stem and vascular bundle count. (**B**) Stem height (black) and stem fresh weight (gray). (**C**) Internode length (black) and stem area (gray). (**D**) Inner (black circle) and outer (black square) vascular bundle area, and interfascicular region area (gray diamond). (**E**) Cell wall thickness of xylem vessel and adjacent bundle fibers of inner (black circle) and outer (black square) vascular bundles, sclerenchyma nearest (gray diamond) and second nearest (gray triangle) to the bundle sheath. Growth stages correspond to elongation (E), inflorescence emergence (F), and senescence (S). Data are means ± standard deviation. Points annotated with the same letter are not significantly different at *P* < 0.05.

We selected three developmental stages to characterize internode and vascular development in *B. distachyon*: elongation, inflorescence emergence, and senescence. For all three stages, the first stem internode above the crown was sampled. During the first developmental stage sampled, the first and only internode was elongating at the base of the tallest tiller (Fig. 1A-B). This was the first appearance of stem tissue, which occurred 18 to 22 days after germination. At the second stage, the inflorescence first emerged from the flag leaf sheath (Fig. 1C-D). This developmental stage occurred 25 to 30 days after germination. These first two stages are roughly equivalent to stages 30 and 51 of the BBCH-scale for cereals [21]. The third stage sampled occurred when the plants had reached their maximum height, and the first internode and basal leaves were completely senesced (Fig. 1E-F). This stage, which occurred 39 to 45 days post germination, is more difficult to equate with the BBCH crop phenology system as it describes grain development characteristics at senescence with developmental stages. The number of stems per plant from the elongation stage to senesce did not change (Fig. 2A). Total stem height significantly increased at each stage by nearly 10 cm, and fresh weight significantly increased from 144 mg to 394 mg (Fig. 2B). The length and width of the first internode increased significantly before inflorescences emerged, but not after (Fig. 2C). These three stages encompass nearly the complete development of a stem internode.

**Figure 3:**
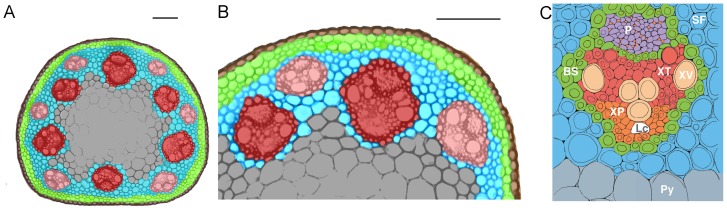
*Brachypodium distachyon* internal stem internode anatomy with emphasis on vasculature. (**A**) Cross section of whole stem and (**B**) higher magnification of the first stem internode. Red, inner vascular bundles; pink, outer vascular bundles; cyan, interfascicular region compromised mostly of sclerenchyma fibers; gray, pith; lime green, chlorenchyma and sclerenchyma cells comprise the cortex; brown, epidermis. (**C**) Vascular bundle illustration at high magnification. Green, bundle sheath (BS); purple, phloem (P); vermilion, companion cells; tan, xylem vessels (XV); red, xylem tracheids (XT); white, lacuna (Lc); orange, xylem parenchyma cells (XP); gray, parenchyma cells (Py); blue, sclerenchyma fibers (SF). (**A-B**) Bar  =  0.1 mm, (**C**) bar  =  0.01 mm.

We characterized numerous anatomical features of the stem at all three developmental stages. *Brachypodium distachyon* possesses an atactostele arrangement of vascular bundles with two circles at the periphery of the stem (Fig. 3A). The innermost vascular bundles are considerably larger than the outer ring of bundles. The cell types within the vascular bundles exhibit a pattern typical of other grasses with the phloem at the outside, and the xylem oriented towards the center of the stem (Fig. 3C). Each vascular bundle is surrounded by bundle sheath cells that also separate the xylem and phloem. The xylem is comprised of large vessels located at the interior end and both sides of the bundle with tracheids located in between the vessels. While not very pronounced, some bundles have a lacuna, which is a region of variable size and shape where protoxylem may have existed. The areas in between the vascular bundles, also known as the interfascicular region, are mostly comprised of sclerenchyma fibers (Fig. 3B). These fibers, along with chlorenchyma, are also located in the layer of cells between the epidermis and outer vascular bundle, a region referred to as the cortex. The center of the stem, a region known as the pith, is populated with parenchyma cells that are larger than surrounding cell types (Fig. 3B). A few parenchyma cells are located in the interfascicular region since the outermost exterior of the pith can be located in between inner vascular bundles (Fig. 3B). Interestingly, all of the vascular bundles were formed at or before the point of elongation, as the number did not significantly change (Fig. 2A). Similarly, the size of the vascular bundles did not change over time with an average size of 3100 µm^2^ for outer bundles and 7800 µm^2^ for inner bundles (Fig. 2D). The area between the vascular bundles significantly increased by 5300 µm^2^ between elongation and inflorescence emergence (Fig. 2D). Therefore, an increase in interfascicular region size and not a change in vascular bundle size accounted for the increase stem internode area before flowering.

### Cell wall deposition in the stem occurs between elongation and senescence

**Figure 4:**
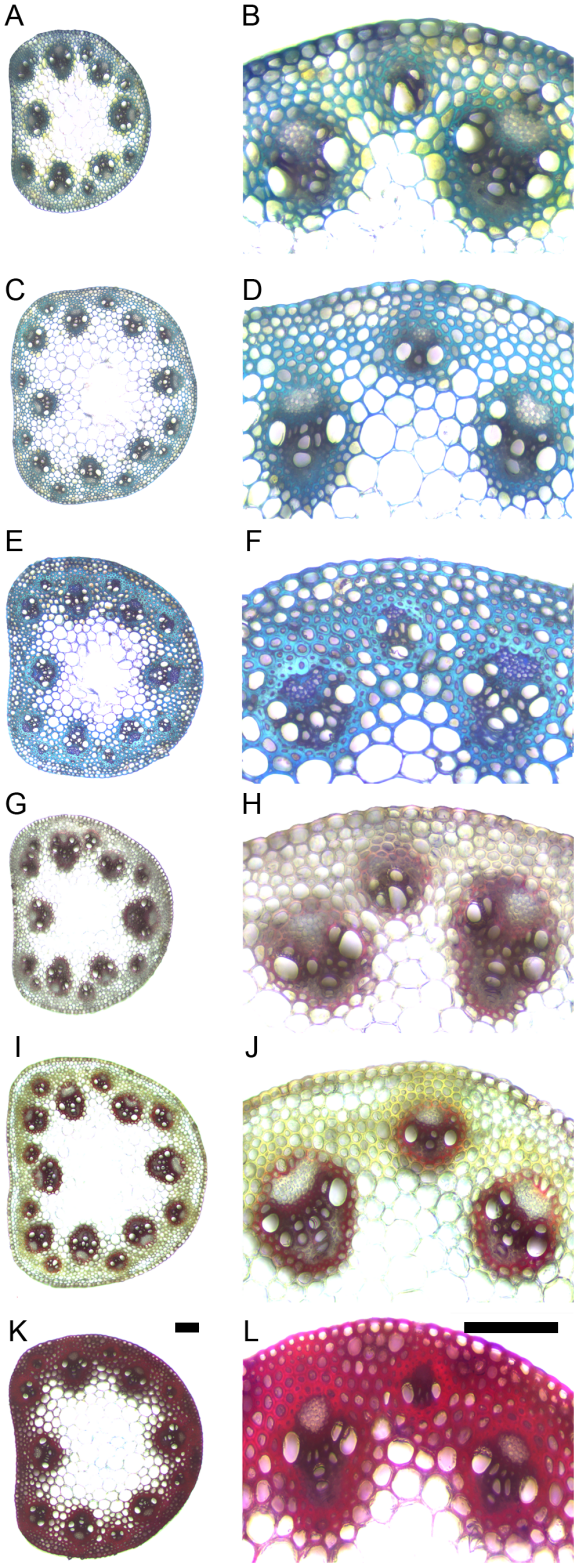
Cell wall thickness and lignin detection increases following stem internode elongation. Whole stem (**A, C, E, G, I, K**) and higher magnification (**B, D, F, H, J, L**) of *Brachypodium distachyon* cross-sections stained with toluidine-blue (**A-F**) or the Wiesner reagent (**G-L**). (**A-B, G-H**) Elongating, (**C-D, I-J**) inflorescence emergence, and (**E-F, K-L**) senesced stem internode transverse cross-sections. Images were taken using brightfield microscopy. Scale bars  =  0.1 mm.

First internode cross-sections were treated with a polychromatic dye, toluidine-blue, that stains polysaccharides violet and lignin turquoise. At elongation, the vascular bundle cells appeared darker and had thicker cell walls than other cell types (Fig. 4A-B). At inflorescence emergence, the vascular bundles and interfascicular region appeared slightly darker and had noticeably thickened cell walls (Fig. 4C-D). At senescence, these same cell types appeared darker, and their cell walls were substantially thicker (Fig. 4E-F). For both inner and outer vascular bundles, the total cell wall size of xylem vessels and adjacent bundle sheath significantly increased between elongation and senescence (Fig. 2E). This growth was quite dramatic with cell wall thickness increasing from 2.7 to 4.6 µm for inner vascular bundles and from 2.0 to 3.3 µm for outer vascular bundles. For the interfascicular region, wall thickness of neighboring sclerenchyma fibers also increased significantly between elongation and senescence (Fig. 2E). The sclerenchyma fibers closest to the bundle sheath thickened from 1.7 to 5.0 µm while the sclerenchyma fibers second nearest to the bundle thickened from 1.7 to 4.1 µm. Thus, cell wall deposition appears to increase following elongation and inflorescence emergence in both the vascular bundle and interfascicular region.

### Lignin deposition increased between stem elongation and senescence

Stem cross-sections were stained with the Wiesner reagent to observe changes in lignin. The Wiesner reagent stains low concentrations of lignin yellow and becomes increasingly red at higher concentrations. At elongation, the vascular bundles stained red while the interfascicular region appeared yellow (Fig. 4G-H). At inflorescence emergence, the vascular bundles stained a more distinctive red and the interfascicular region a darker yellow (Fig. 4I-J). At senescence, the vascular bundles and interfascicular region both stained a dark red (Fig. 4K-L).

**Figure 5:**
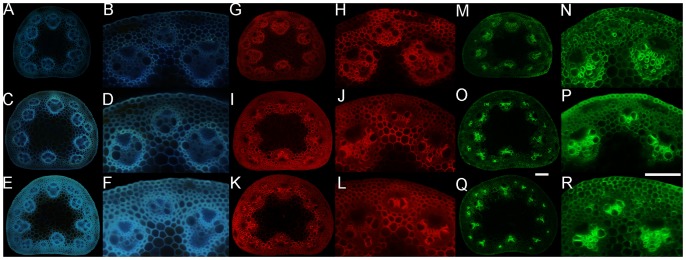
Florescent detection of lignin and indirect immunodetection of crystalline cellulose and xylan in stem internode. Whole stem (**A, C, E, G, I, K, M, O, Q**) and higher magnification (**B, D, F, H, J, L, N, P, R**) of *Brachypodium distachyon* cross sections observing lignin autofluorescence (**A-F**) and immunolabeled CBM3a probe (**G-L**) and LM10 antibody (**M-R**). (**A, B, G, H, M, N**) Elongating, (**C, D, I, J, O, P**) inflorescence emergence, and (**E, F, K, L, Q, R**) senesced stem internode transverse cross-sections. Images were taken using wide field epifluorescence microscopy. Scale bars  =  0.1 mm.

**Figure 6:**
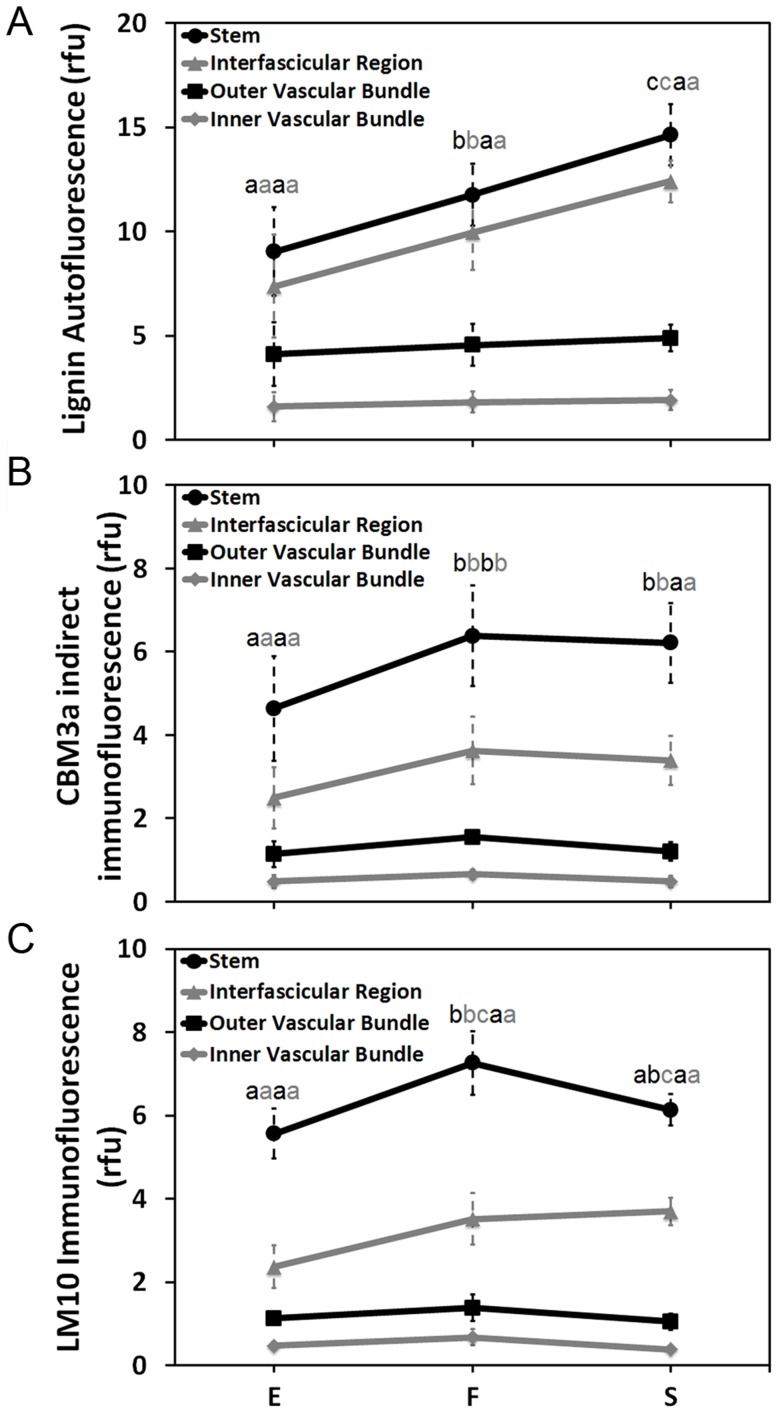
Quantification of florescence detection of lignin and indirect immunodetection of crystalline cellulose and xylan in stem internode. Corrected (**A**) total autofluorescence, (**B**) CBM3a indirect immunofluorescence, and (**C**) LM10 immunofluorescence of whole stem (back circle), inner (gray diamond) and outer (black square) vascular bundles, and interfascicular region (gray triangle). Data are means ± standard deviation. Points annotated with the same letter are not significantly different at *P* < 0.05.

As phenolic groups fluoresce, fluorescence microscopy was used as relative estimate of lignin content within stems. In elongating internodes, the vascular bundles were visible at low magnification whereas the interfascicular region could only be detected at higher magnifications (Fig. 5A-B). At inflorescence emergence, the interfascicular region was noticeably more fluorescent (Fig. 5C-D). At senescence, the vascular bundles were bright and the interfascicular region became strikingly more fluorescent (Fig. 5E-F). We then quantified the total corrected lignin autofluorescence of the whole stem, vascular bundles, and interfascicular region. The whole stem significantly increased in total autofluorescence between each stage sampled (Fig. 6A). Both inner and outer vascular bundles increased only slightly in total autofluorescence over time (Fig. 6A). The interfascicular region had the most substantial change in total autofluorescence where it increased by nearly two-fold between elongation and senescence (Fig. 6A). These observations demonstrate that significant lignin deposition occurred following elongation and inflorescence emergence in the interfascicular region, but not the vascular bundles.

### Crystalline cellulose and xylan deposition increased between stem elongation and inflorescence emergence

Stem cross-sections were immunolabeled with the CBM3a recombinant protein probe in order to observe crystalline cellulose. At elongation, the xylem was more fluorescent than the interfascicular region (Fig. 5G-H). The interfascicular region was more apparent at inflorescence emergence but the vascular bundle fluorescence did not increase (Fig. 5I-J). Interestingly, neither the interfascicular region nor the vascular bundles seemed any more fluorescent at senescence (Fig. 5K-L). We then quantified the total corrected crystalline cellulose fluorescence of the whole stem, vascular bundles, and interfascicular region. The total crystalline cellulose fluorescence significantly increased between elongation and inflorescence emergence whereupon it appeared to plateau (Fig. 6B). This same trend was observed for inner vascular bundles and the interfascicular region plateaued, while both vascular bundle types interestingly decreased slightly (Fig. 6B). For outer vascular bundles, there was no change in fluorescence after internode elongation (Fig. 6B). Thus, crystalline cellulose immunolabeling increased in vascular bundles and the interfascicular region between elongation and inflorescence emergence and then seemingly ceased afterwards. To observe changes in xylan content, stem sections were immunolabelled with a monoclonal antibody against xylan. The total stem and interfascicular region fluorescence was greater at inflorescence emergence than elongation (Fig. 5M-P, 6C). In contrast, fluorescence did not significantly increase in the vascular bundles following elongation (Fig. 5O-R, 6C). Interestingly, total stem florescence significantly decreased between inflorescence emergence and senescence. Unlike the CBM3a indirect immunofluorescence detection of crystalline cellulose, the concentration of immunofluorescence detected xylan was appreciably greater in the xylem cells than the interfascicular fibers while their detection were noticeably difficult to observe in the bundle sheath.

### Changes in cell wall associated FTIR spectra in developing stem internodes

**Figure 7:**
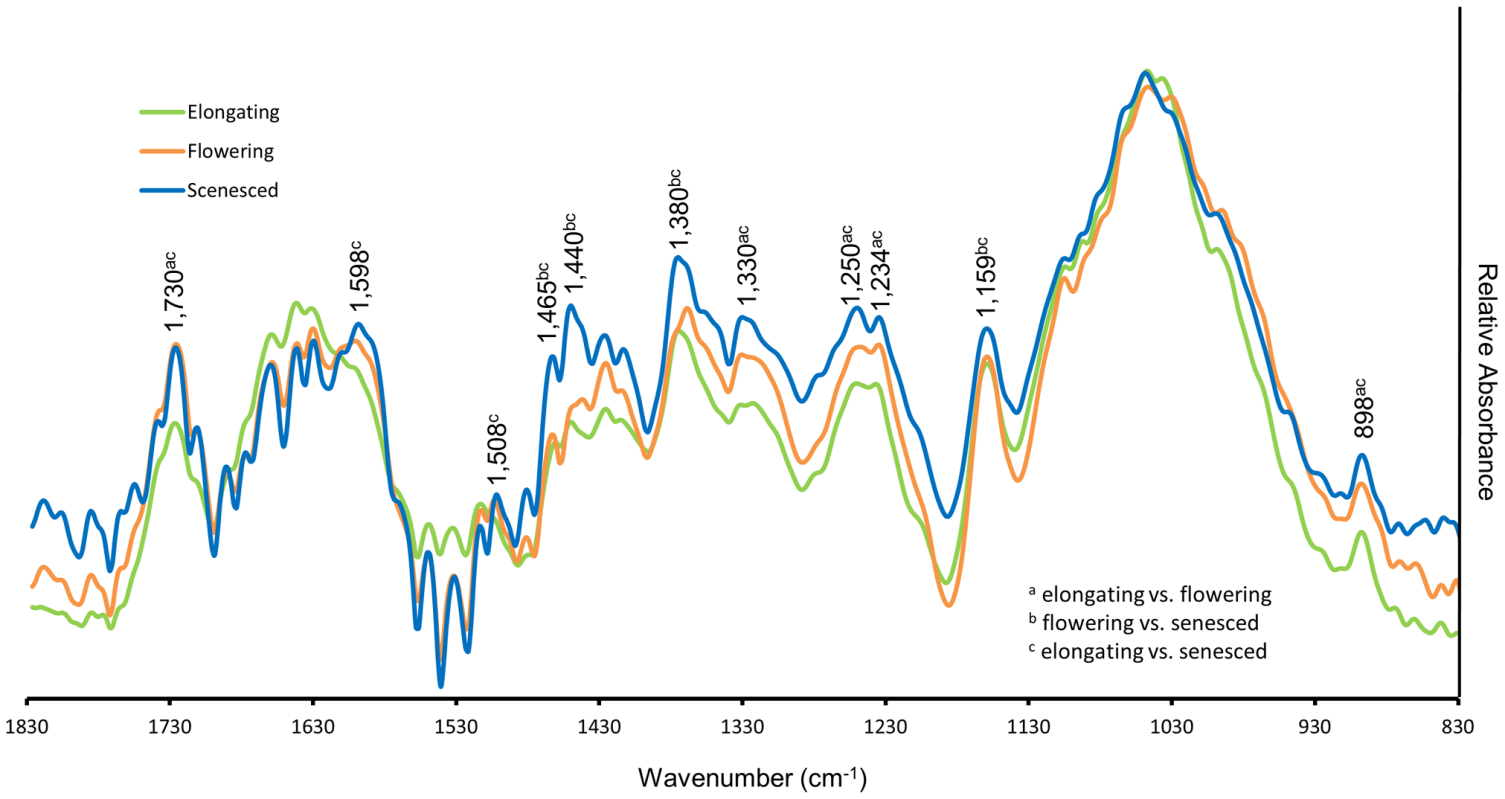
Characterization of wall composition changes associated with growth using FTIR spectroscopy. Average line spectra of stem tissue sampled from elongating (green), flowering (orange), and senesced (blue) stages of development. Wavenumbers corresponding to absorbance peaks associated with cellulose, hemicellulose, and lignin are noted. Letters indicate significant differences at *P* < 0.05.

The complex and generally hydrophobic secondary wall matrix can be difficult to penetrate with histo- and immunochemical reagents. In an attempt to better quantify cell wall modifications that occurred during stem development, ground tissue obtained from basal internodes was analyzed by FTIR spectroscopy. Typical spectra were obtained for all samples and the relative absorbance at wavenumbers associated with cell wall components were generally greater as the stems matured (Fig. 7). Peak absorbance at 1440 (O-H in-plane bending; [22]), 1380 (C-H bending; [22], [23], [24]) and 1330 cm^−1^ (C–H vibration and O–H in-plane bending; [23], [25]) associated collectively with lignin, hemicelluloses, and cellulose consistently increased as the stem developed. Wavenumbers specific to lignin were significantly greater in senesced than elongating stems. These include C-H deformation at 1465 cm^−1^ and aromatic ring vibrations at 1598 and 1508 cm^−1^ [23], [26]. Spectra for senesced stem internodes were also significantly greater for cellulose at 1159 and 898 cm^−1^ [22], [27], [28]. Peaks observed at 1,234 and 1,250 cm^−1^ (C = O stretching; [25], [27]) may represent crystalline cellulose; however, further investigation is needed to verify this possibility. Consistent with the LM10 immunolabeling, peak absorbance at 1730 cm^−1^ (C = O stretching; [23], [26], [29], [30]) suggests significant xylan accumulation from elongation to inflorescence emergence. Together, the FTIR absorbances at wavenumbers assigned to cellulose, hemicellulose, and lignin were consistent with the immunohistochemistry results that demonstrate changes in cell wall deposition following stem elongation.

## Discussion

Here we describe and quantify growth and cell wall accumulation of three stages of *B. distachyon* stem internode development. Samples were collected at distinct developmental stages rather than time after germination. Interestingly, the size and number of vascular bundles did not change after the point of stem elongation. Therefore, the increase in stem internode area was due to changes in the interfascicular region. On the other hand, changes in fresh weight were possibly the result of secondary cell wall thickening, which again, did not result in a change in vascular bundle size. While we cannot rule out changes at earlier stages of growth, vascular bundle structure appears to be established at or close to the point of meristem differentiation.

The term for grass vascular patterning is atactostele; atacto meaning disarranged. However, the arrangement is clearly not random and scattered. For example, the two circles of vascular bundles, the inner bundles being considerably larger than the outer, observed in *B. distachyon* are regularly spaced and of fairly predictable number. This design is similar to closely related C_3_ crop species such as *Oryza sativa* and *Triticum aestivum* with some subtle differences [31], [32]. In *O. sativa* and *T. aestivum*, the inner and outer vascular bundles are located directly across from each other and parenchyma cells surround the inner bundles while outer bundles are surrounded by sclerenchyma fibers. Similarly, the forage grasses *Lolium perenne* and *Festuca arundinacea* have two rings of vascular bundles, but they are not consistently oriented across or diagonally to each other [33], [34]. In *B. distachyon*, inner and outer vascular bundles are offset to each other, and both types of bundles are surrounded by sclerenchyma fibers. For this reason, the interfascicular region is mostly comprised of sclerenchyma fibers in *B. distachyon*. Larger grass species such as *Zea mays* and *S. bicolor* have many more vascular bundles with a spiral pattern from the center of the stem [1], [35], [36]. The rind is the region near the epidermis with small vascular bundles surrounded by highly thickened sclerenchyma fibers. This area has all the hallmarks of the interfascicular region of *B. distachyon*. In C_4_ grasses, leaf bundle sheath cells contain chloroplast and thin walls [37]. We use the term bundle sheath as an anatomical term, rather than a functional label [1]. In *B. distachyon*, these cells have thick walls and lack chloroplasts. The cell types and organization of the ground tissue outside of vascular bundles are typical of other monocots. Chlorenchyma cells are found within the stem adjacent to the epidermis and collenchyma cells are not observed in the cortex, an observation typical of grasses.

The vascular bundle and interfascicular region walls continued to thicken and thus cell wall deposition continued following internode elongation and inflorescence emergence. Previous transcriptomic analysis at the resolution of whole tissue types revealed a strong correlation between lignin and cellulose structural gene expression [38], [39]. Histological observation revealed differences in both the temporal and spatial patterns of lignin and crystalline cellulose deposition. If we infer wall composition by histological quantification, lignin deposition increased between stem internode elongation and senescence in sclerenchyma fibers and tracheary elements, cells known to undergo extensive secondary cell wall biosynthesis [40]. Increased lignification was also observed in parenchyma cells, which do not undergo secondary cell wall thickening. While primary cell walls are often not considered lignified, this observation is not unusual [33], [34], [41]. Unlike lignin, crystalline cellulose and xylan immunolabeling did not increase in the first internode following inflorescence emergence and was most abundant in xylem vessels and tracheids. While relatively little crystalline cellulose and xylan immunolabeling was observed in the bundle sheath cells, they were heavily lignified which might have limited access of the CBM3a probe to bind crystalline cellulose or the LM10 antibody to bind xylan. This could explain why a slight decrease of crystalline cellulose and xylan detection was observed in vascular bundles between inflorescence emergence and senescence.

Xylem cells undergo apoptosis before becoming functional water transporters [42]. As the cells mature, secondary wall formation occurs concurrent with the vacuolar accumulation of autolytic factors associated with cell death [43]. After a critical level of serine protease is reached in the extracellular space, an influx of calcium ions into the cytoplasm triggers a rupture of the central vacuole releasing the autolytic factors that lead to rapid cell death [43], [44]. Remarkably, even after death, the cell walls of tracheary elements continue to lignify [45]. Although this process is not well understood, xylem parenchyma cells may deposit lignin monomers into the walls of dead cells and provide the hydrogen peroxide needed for polymerization [46], [47]. In developing *B. distachyon* stem internode, cell death in the first internode likely occurs following the biosynthesis of cellulose, sometime before inflorescence emergence. After this point, lignin deposition in xylem and fiber walls continued. These mechanisms could explain the differential detection of crystalline cellulose and lignin following internode elongation since lignification would continue even after cellulose and lignin deposition have concluded.

## Supporting Information

Figure S1 Nominal autofluorescence was observed when analyzing unlabeled sections under Texas Red filter. Stem of *Brachypodium distachyon* unlabeled transverse section using Nomarski optics (**A**), and unlabeled (**B**), immunolabeled CBM3a probe (**C**), and LM10 antibody (**D**) using Texas Red 560 - 540 nm filter. All florescent images were taken using wide field epifluorescence microscopy with identical acquisition settings. Scale bars  =  0.1 mm.
(TIF)
