# Supplementary figures and images for: Cell Walls and the Developmental Anatomy of the Brachypodium distachyon Stem Internode
Source: PLoS One. 2013 Nov 21;8(11):e80640. doi: 10.1371/journal.pone.0080640 (PMC3836760; doi:10.1371/journal.pone.0080640)

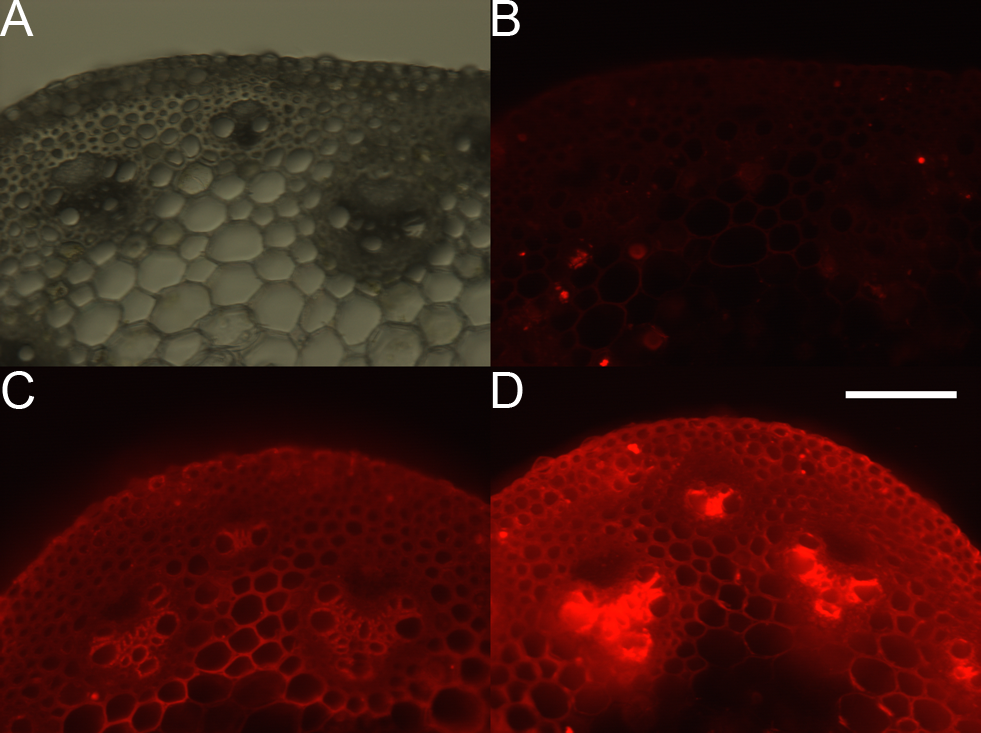

Supplement: Figure S1 — Nominal autofluorescence was observed when analyzing unlabeled sections under Texas Red filter. Stem of Brachypodium distachyon unlabeled transverse section using Nomarski optics (A), and unlabeled (B), immunolabeled CBM3a probe (C), and LM10 antibody (D) using Texas Red 560 - 540 nm filter. All florescent images were taken using wide field epifluorescence microscopy with identical acquisition settings. Scale bars = 0.1 mm. (TIF) [file pone.0080640.s001.tif]
